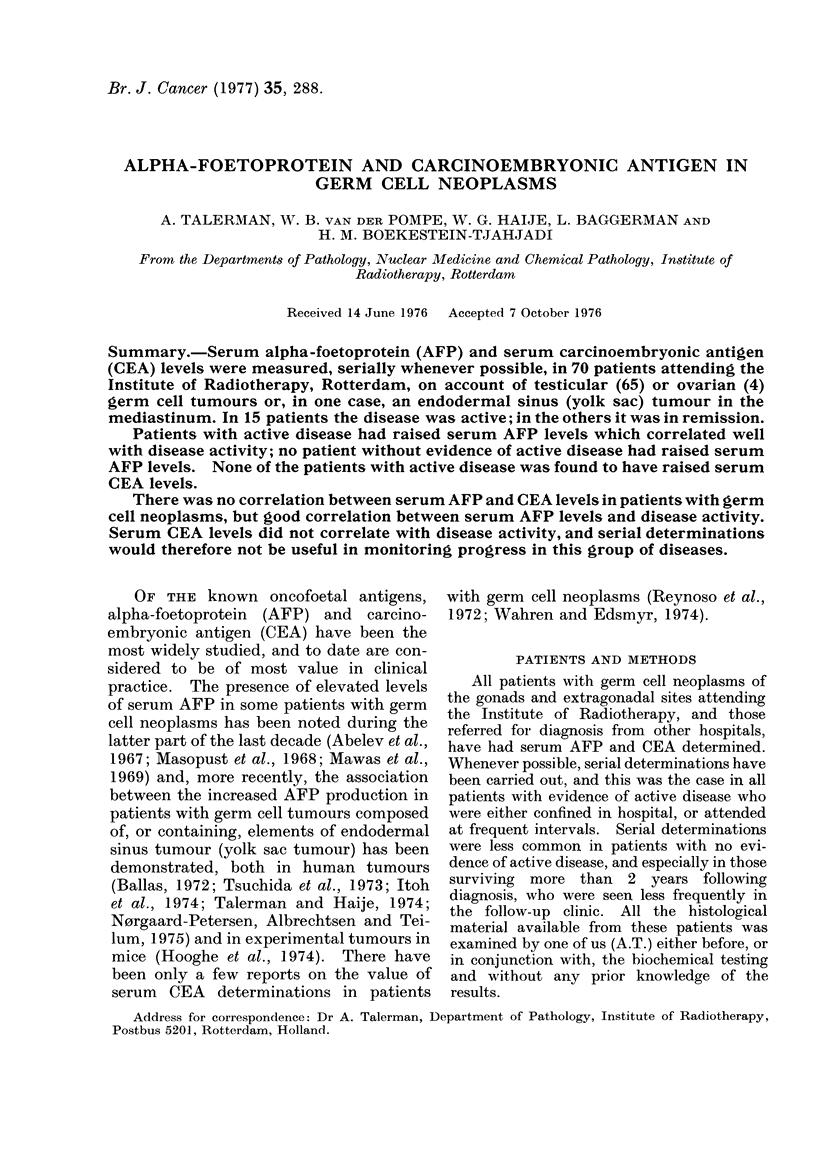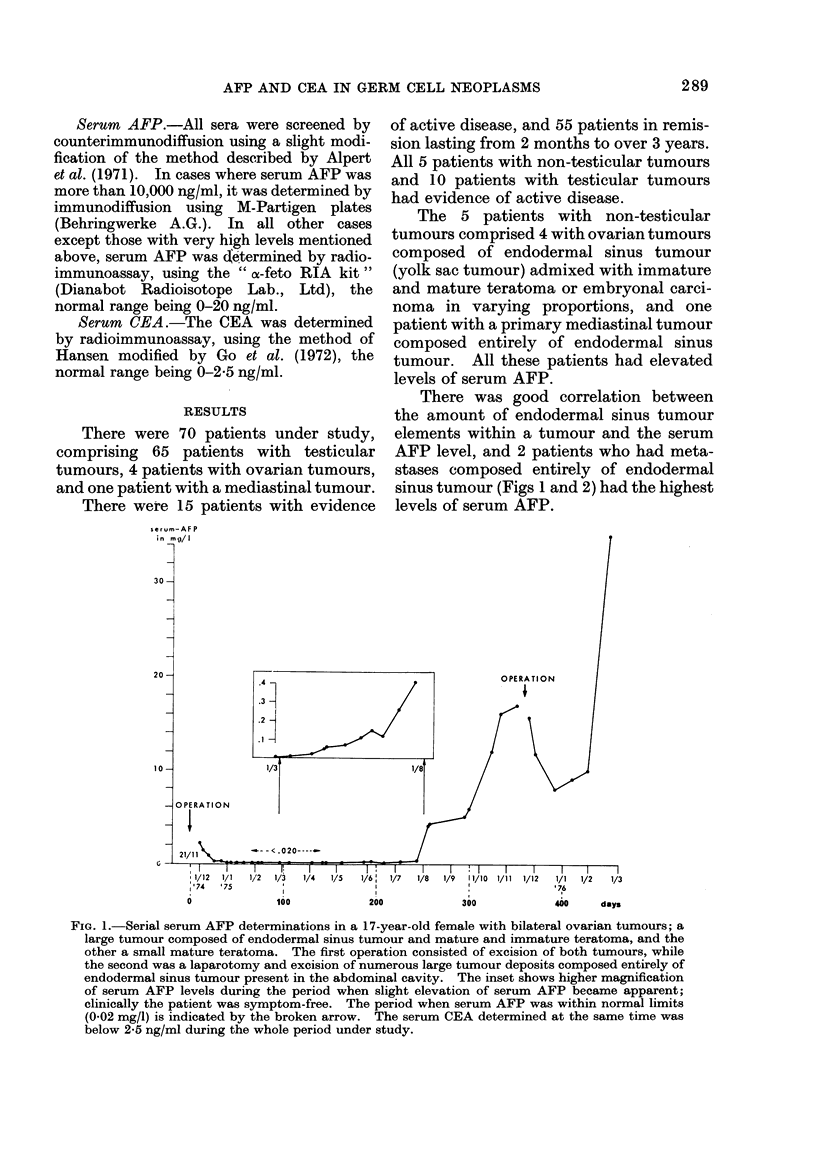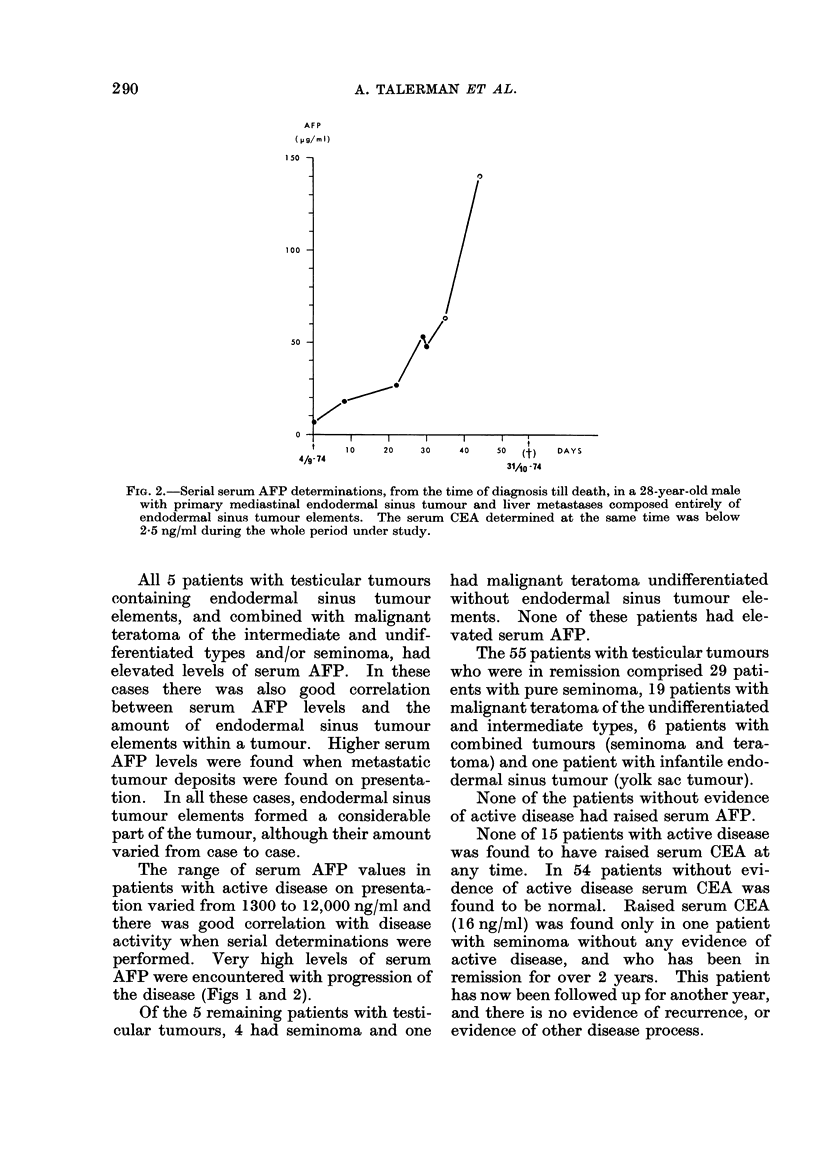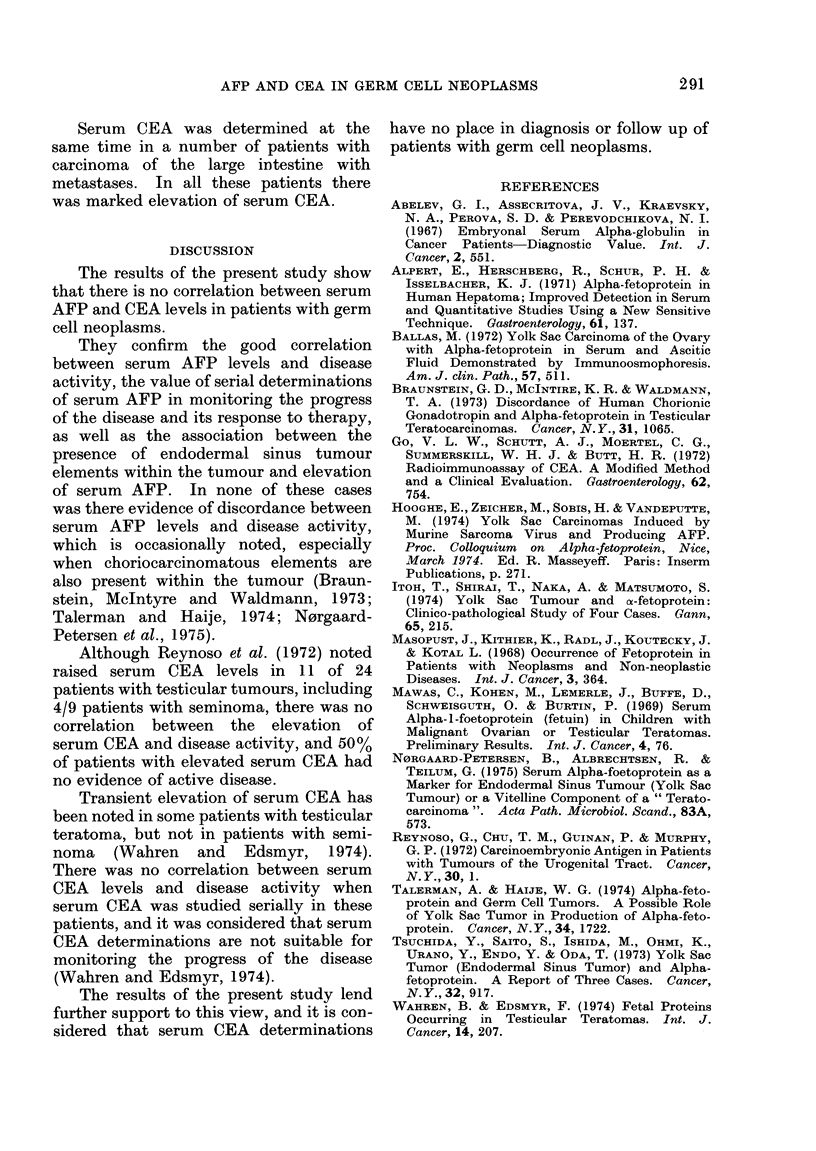# Alpha-foetoprotein and carcinoembryonic antigen in germ cell neoplasms.

**DOI:** 10.1038/bjc.1977.41

**Published:** 1977-03

**Authors:** A. Talerman, W. B. van der Pompe, W. G. Haije, L. Baggerman, H. M. Boekestein-Tjahjadi

## Abstract

Serum alpha-foetoprotein (AFP) and serum carcinoembryonic antigen (CEA) levels were measured, serially whenever possible, in 70 patients attending the Institute of Radiotherapy, Rotterdam, on account of testicular (65) or ovarian (4) germ cell tumours or, in one case, an endodermal sinus (yolk sac) tumour in the mediastinum. In 15 patients the disease was active; in the others it was in remission. Patients with active disease had raised serum AFP levels which correlated well with disease activity; no patient without evidence of active disease had raised serum AFP levels. None of the patients with active disease was found to have raised serum CEA levels. There was no correlation between serum AFP and CEA levels in patients with germ cell neoplasms, but good correlation between serum AFP levels and disease activity. Serum CEA levels did not correlate with disease activity, and serial determinations would therefore not be useful in monitoring progress in this group of diseases.


*Br. J. Cancer* (1977) **35**, 288.

### ALPHA-FOETOPROTEIN AND CARCINOEMBRYONIC ANTIGEN IN GERM CELL NEOPLASMS

A. TALERMAN, W. B. VAN DER POMPE, W. G. HAIJE, L. BAGGERMAN AND H. M. BOEKESTEIN-TJAHJADI

*From the Departments of Pathology, Nuclear Medicine and Chemical Pathology, Institute of Radiotherapy, Rotterdam*

Received 14 June 1976 Accepted 7 October 1976

**Summary.—Serum alpha-foetoprotein (AFP) and serum carcinoembryonic antigen (CEA) levels were measured, serially whenever possible, in 70 patients attending the Institute of Radiotherapy, Rotterdam, on account of testicular (65) or ovarian (4) germ cell tumours or, in one case, an endodermal sinus (yolk sac) tumour in the mediastinum. In 15 patients the disease was active; in the others it was in remission.**

**Patients with active disease had raised serum AFP levels which correlated well with disease activity; no patient without evidence of active disease had raised serum AFP levels. None of the patients with active disease was found to have raised serum CEA levels.**

**There was no correlation between serum AFP and CEA levels in patients with germ cell neoplasms, but good correlation between serum AFP levels and disease activity. Serum CEA levels did not correlate with disease activity, and serial determinations would therefore not be useful in monitoring progress in this group of diseases.**

OF THE known oncofoetal antigens, alpha-foetoprotein (AFP) and carcinoembryonic antigen (CEA) have been the most widely studied, and to date are considered to be of most value in clinical practice. The presence of elevated levels of serum AFP in some patients with germ cell neoplasms has been noted during the latter part of the last decade (Abelev *et al.*, 1967; Masopust *et al.*, 1968; Mawas *et al.*, 1969) and, more recently, the association between the increased AFP production in patients with germ cell tumours composed of, or containing, elements of endodermal sinus tumour (yolk sac tumour) has been demonstrated, both in human tumours (Ballas, 1972; Tsuchida *et al.*, 1973; Itoh *et al.*, 1974; Talerman and Haije, 1974; Nørgaard-Petersen, Albrechtsen and Teilum, 1975) and in experimental tumours in mice (Hooghe *et al.*, 1974). There have been only a few reports on the value of serum CEA determinations in patients with germ cell neoplasms (Reynoso *et al.*, 1972; Wahren and Edsmyr, 1974).

### PATIENTS AND METHODS

All patients with germ cell neoplasms of the gonads and extragonadal sites attending the Institute of Radiotherapy, and those referred for diagnosis from other hospitals, have had serum AFP and CEA determined. Whenever possible, serial determinations have been carried out, and this was the case in all patients with evidence of active disease who were either confined in hospital, or attended at frequent intervals. Serial determinations were less common in patients with no evidence of active disease, and especially in those surviving more than 2 years following diagnosis, who were seen less frequently in the follow-up clinic. All the histological material available from these patients was examined by one of us (A.T.) either before, or in conjunction with, the biochemical testing and without any prior knowledge of the results.

Address for correspondence: Dr A. Talerman, Department of Pathology, Institute of Radiotherapy, Postbus 5201, Rotterdam, Holland.

*Serum AFP.*—All sera were screened by counterimmunodiffusion using a slight modification of the method described by Alpert *et al.* (1971). In cases where serum AFP was more than 10,000 ng/ml, it was determined by immunodiffusion using M-Partigen plates (Behringwerke A.G.). In all other cases except those with very high levels mentioned above, serum AFP was determined by radioimmunoassay, using the " α-feto RIA kit " (Dianabot Radioisotope Lab., Ltd), the normal range being 0–20 ng/ml.

*Serum CEA.*—The CEA was determined by radioimmunoassay, using the method of Hansen modified by Go *et al.* (1972), the normal range being 0–2·5 ng/ml.

### RESULTS

There were 70 patients under study, comprising 65 patients with testicular tumours, 4 patients with ovarian tumours, and one patient with a mediastinal tumour.

There were 15 patients with evidence of active disease, and 55 patients in remission lasting from 2 months to over 3 years. All 5 patients with non-testicular tumours and 10 patients with testicular tumours had evidence of active disease.

The 5 patients with non-testicular tumours comprised 4 with ovarian tumours composed of endodermal sinus tumour (yolk sac tumour) admixed with immature and mature teratoma or embryonal carcinoma in varying proportions, and one patient with a primary mediastinal tumour composed entirely of endodermal sinus tumour. All these patients had elevated levels of serum AFP.

There was good correlation between the amount of endodermal sinus tumour elements within a tumour and the serum AFP level, and 2 patients who had metastases composed entirely of endodermal sinus tumour (Figs 1 and 2) had the highest levels of serum AFP.

FIG. 1.—Serial serum AFP determinations in a 17-year-old female with bilateral ovarian tumours; a large tumour composed of endodermal sinus tumour and mature and immature teratoma, and the other a small mature teratoma. The first operation consisted of excision of both tumours, while the second was a laparotomy and excision of numerous large tumour deposits composed entirely of endodermal sinus tumour present in the abdominal cavity. The inset shows higher magnification of serum AFP levels during the period when slight elevation of serum AFP became apparent; clinically the patient was symptom-free. The period when serum AFP was within normal limits (0·02 mg/l) is indicated by the broken arrow. The serum CEA determined at the same time was below 2·5 ng/ml during the whole period under study.

FIG. 2.—Serial serum AFP determinations, from the time of diagnosis till death, in a 28-year-old male with primary mediastinal endodermal sinus tumour and liver metastases composed entirely of endodermal sinus tumour elements. The serum CEA determined at the same time was below 2·5 ng/ml during the whole period under study.

All 5 patients with testicular tumours containing endodermal sinus tumour elements, and combined with malignant teratoma of the intermediate and undifferentiated types and/or seminoma, had elevated levels of serum AFP. In these cases there was also good correlation between serum AFP levels and the amount of endodermal sinus tumour elements within a tumour. Higher serum AFP levels were found when metastatic tumour deposits were found on presentation. In all these cases, endodermal sinus tumour elements formed a considerable part of the tumour, although their amount varied from case to case.

The range of serum AFP values in patients with active disease on presentation varied from 1300 to 12,000 ng/ml and there was good correlation with disease activity when serial determinations were performed. Very high levels of serum AFP were encountered with progression of the disease (Figs 1 and 2).

Of the 5 remaining patients with testicular tumours, 4 had seminoma and one had malignant teratoma undifferentiated without endodermal sinus tumour elements. None of these patients had elevated serum AFP.

The 55 patients with testicular tumours who were in remission comprised 29 patients with pure seminoma, 19 patients with malignant teratoma of the undifferentiated and intermediate types, 6 patients with combined tumours (seminoma and teratoma) and one patient with infantile endodermal sinus tumour (yolk sac tumour).

None of the patients without evidence of active disease had raised serum AFP.

None of 15 patients with active disease was found to have raised serum CEA at any time. In 54 patients without evidence of active disease serum CEA was found to be normal. Raised serum CEA (16 ng/ml) was found only in one patient with seminoma without any evidence of active disease, and who has been in remission for over 2 years. This patient has now been followed up for another year, and there is no evidence of recurrence, or evidence of other disease process.

Serum CEA was determined at the same time in a number of patients with carcinoma of the large intestine with metastases. In all these patients there was marked elevation of serum CEA.

### DISCUSSION

The results of the present study show that there is no correlation between serum AFP and CEA levels in patients with germ cell neoplasms.

They confirm the good correlation between serum AFP levels and disease activity, the value of serial determinations of serum AFP in monitoring the progress of the disease and its response to therapy, as well as the association between the presence of endodermal sinus tumour elements within the tumour and elevation of serum AFP. In none of these cases was there evidence of discordance between serum AFP levels and disease activity, which is occasionally noted, especially when choriocarcinomatous elements are also present within the tumour (Braunstein, McIntyre and Waldmann, 1973; Talerman and Haije, 1974; Nørgaard-Petersen *et al.*, 1975).

Although Reynoso *et al.* (1972) noted raised serum CEA levels in 11 of 24 patients with testicular tumours, including 4/9 patients with seminoma, there was no correlation between the elevation of serum CEA and disease activity, and 50% of patients with elevated serum CEA had no evidence of active disease.

Transient elevation of serum CEA has been noted in some patients with testicular teratoma, but not in patients with seminoma (Wahren and Edsmyr, 1974). There was no correlation between serum CEA levels and disease activity when serum CEA was studied serially in these patients, and it was considered that serum CEA determinations are not suitable for monitoring the progress of the disease (Wahren and Edsmyr, 1974).

The results of the present study lend further support to this view, and it is considered that serum CEA determinations have no place in diagnosis or follow up of patients with germ cell neoplasms.

### REFERENCES

ABELEV, G. I., ASSECRITOVA, J. V., KRAEVSKY, N. A., PEROVA, S. D. & PEREVODCHIKOVA, N. I. (1967) Embryonal Serum Alpha-globulin in Cancer Patients—Diagnostic Value. *Int. J. Cancer*, **2**, 551.

ALPERT, E., HERSCHBERG, R., SCHUR, P. H. & ISSELBACHER, K. J. (1971) Alpha-fetoprotein in Human Hepatoma; Improved Detection in Serum and Quantitative Studies Using a New Sensitive Technique. *Gastroenterology*, **61**, 137.

BALLAS, M. (1972) Yolk Sac Carcinoma of the Ovary with Alpha-fetoprotein in Serum and Ascitic Fluid Demonstrated by Immunoosmophoresis. *Am. J. clin. Path.*, **57**, 511.

BRAUNSTEIN, G. D., MCINTIRE, K. R. & WALDMANN, T. A. (1973) Discordance of Human Chorionic Gonadotropin and Alpha-fetoprotein in Testicular Teratocarcinomas. *Cancer, N.Y.*, **31**, 1065.

GO, V. L. W., SCHUTT, A. J., MOERTEL, C. G., SUMMERSKILL, W. H. J. & BUTT, H. R. (1972) Radioimmunoassay of CEA. A Modified Method and a Clinical Evaluation. *Gastroenterology*, **62**, 754.

HOOGHE, E., ZEICHER, M., SOBIS, H. & VANDEPUTTE, M. (1974) Yolk Sac Carcinomas Induced by Murine Sarcoma Virus and Producing AFP. *Proc. Colloquium on Alpha-fetoprotein, Nice, March 1974.* Ed. R. Masseyeff. Paris: Inserm Publications, p. 271.

ITOH, T., SHIRAI, T., NAKA, A. & MATSUMOTO, S. (1974) Yolk Sac Tumour and $\alpha$-fetoprotein: Clinico-pathological Study of Four Cases. *Gann*, **65**, 215.

MASOPUST, J., KITHIER, K., RADL, J., KOUTECKY, J. & KOTAL L. (1968) Occurrence of Fetoprotein in Patients with Neoplasms and Non-neoplastic Diseases. *Int. J. Cancer*, **3**, 364.

MAWAS, C., KOHEN, M., LEMERLE, J., BUFFE, D., SCHWEISGUTH, O. & BURTIN, P. (1969) Serum Alpha-1-foetoprotein (fetuin) in Children with Malignant Ovarian or Testicular Teratomas. Preliminary Results. *Int. J. Cancer*, **4**, 76.

NØRGAARD-PETERSEN, B., ALBRECHTSEN, R. & TEILUM, G. (1975) Serum Alpha-foetoprotein as a Marker for Endodermal Sinus Tumour (Yolk Sac Tumour) or a Vitelline Component of a " Teratocarcinoma ". *Acta Path. Microbiol. Scand.*, **83A**, 573.

REYNOSO, G., CHU, T. M., GUINAN, P. & MURPHY, G. P. (1972) Carcinoembryonic Antigen in Patients with Tumours of the Urogenital Tract. *Cancer, N.Y.*, **30**, 1.

TALERMAN, A. & HAIJE, W. G. (1974) Alpha-fetoprotein and Germ Cell Tumors. A Possible Role of Yolk Sac Tumor in Production of Alpha-fetoprotein. *Cancer, N.Y.*, **34**, 1722.

TSUCHIDA, Y., SAITO, S., ISHIDA, M., OHMI, K., URANO, Y., ENDO, Y. & ODA, T. (1973) Yolk Sac Tumor (Endodermal Sinus Tumor) and Alphafetoprotein. A Report of Three Cases. *Cancer, N.Y.*, **32**, 917.

WAHREN, B. & EDSMYR, F. (1974) Fetal Proteins Occurring in Testicular Teratomas. *Int. J. Cancer*, **14**, 207.